# Prognostic Value of the TLM3 Biomarker Panel for Early Fibrosis Development in MASLD Within the General Population

**DOI:** 10.1111/liv.70169

**Published:** 2025-06-24

**Authors:** Koen C. van Son, Jelle C. B. C. de Jong, Serdar Özsezen, Martien P. M. Caspers, Quinten J. J. Augustijn, Jessica Snabel, Henrike Galenkamp, Henrike M. Hamer, Anne‐Marieke van Dijk, Arianne van Koppen, Anne Linde Mak, Bert‐Jan van den Born, Max Nieuwdorp, Joost P. H. Drenth, Lise Lotte Gluud, Maarten E. Tushuizen, Roeland Hanemaaijer, Lars Verschuren, Adriaan G. Holleboom

**Affiliations:** ^1^ Department of Vascular Medicine Amsterdam UMC, University of Amsterdam Amsterdam the Netherlands; ^2^ Department of Gastroenterology and Hepatology Amsterdam UMC, University of Amsterdam Amsterdam the Netherlands; ^3^ Amsterdam Gastroenterology Endocrinology Metabolism (AGEM) Institute Amsterdam UMC, University of Amsterdam Amsterdam the Netherlands; ^4^ TNO Health & Work Leiden the Netherlands; ^5^ Department of Public and Occupational Health Amsterdam UMC, University of Amsterdam Amsterdam the Netherlands; ^6^ Department of Laboratory Medicine, Laboratory Specialized Diagnostics & Research Amsterdam UMC, University of Amsterdam Amsterdam the Netherlands; ^7^ Department of Gastroenterology and Hepatology, Copenhagen University Hospital University of Copenhagen Copenhagen Denmark; ^8^ Department of Gastroenterology and Hepatology LUMC Leiden the Netherlands

**Keywords:** biomarker, disease progression, fibrogenesis, MASH, non‐invasive

## Abstract

**Background & Aims:**

Fibrotic MASLD is associated with increased morbidity and mortality, often remaining asymptomatic until advanced stages of disease. Predicting fibrosis onset and progression would improve risk stratification and treatment allocation. This study aims to investigate whether a previously identified fibrosis biomarker panel for active fibrogenesis (TLM3) can serve as a prognostic marker panel for fibrosis development in a population at cardiometabolic risk of fibrotic MASLD.

**Methods:**

The temporal dynamics of a molecular fibrosis gene expression signature associated with histologically proven fibrosis development was investigated in a diet‐induced MASLD mouse model (LDLr−/−.Leiden). The corresponding proteins were measured in baseline serum from individuals at risk of MASLD from the general population HELIUS‐cohort and correlated with established fibrosis proxies (ELF, VCTE and FIB4) at 7 years follow‐up.

**Results:**

The molecular fibrosis gene expression signature was upregulated in a murine MASLD model before the onset of histopathological features of fibrosis. In humans, serum levels of IGFBP7, Ssc5D, Sema4D, VCAN, THBS1 and TNC at baseline correlated with fibrosis proxies at follow‐up. IGFBP7 at baseline was able to predict new onset fibrosis, defined as ELF ≥ 9.8 at follow‐up in participants with ELF < 9.8 at baseline, with an area under the curve (AUC) of 0.79 (95% CI: 0.64–0.94).

**Conclusion:**

Together, these findings indicate the potential predictive capacity of the TLM3 biomarker panel in early stages of MASLD‐fibrosis, both in a murine model as well as in individuals from the general population at risk of MASLD.

**Abbreviations**
ADAMTS2: ADAM metallopeptidase with thrombospondin type 1 motif 2
ALT: alanine transaminase
Amsterdam UMC: Amsterdam University Medical Center
APRI: AST to platelet ratio
AST: aspartate transaminase
AUC: area under the curve
BMI: body mass index
CAP: controlled attenuation parameter
CI: confidence interval
CXCL10: C‐X‐C motif chemokine ligand 10
dB/m: decibel per meter
ELF: enhanced liver fibrosis‐test
F3: advanced fibrosis
FBN1: fibrillin‐1
FIB4: fibrosis‐4 score
H&E: haematoxylin and eosin
HA: hyaluronic acid
HbA1c: haemoglobulin A1c
HDL: high‐density lipoprotein
HELIUS study: healthy life in an urban setting study
IGFBP7: insulin‐like growth factor‐binding protein 7
IQR: interquartile range
kPa: kilo Pascals
LDL: low‐density lipoprotein
LSM: liver stiffness measurement
MASH: metabolic dysfunction‐associated steatohepatitis
MASLD: metabolic dysfunction‐associated steatohepatitis
NILE study: NAFLD in healthy life in an urban setting‐study
NITs: non‐invasive tests
OR: odds ratio
PAM: peptidylglycine alpha‐amidating monooxygenase
PIIINP: procollagen III amino terminal peptide
PLAU: plasminogen activator
SD: standard deviation
Sema4D: semaphorin‐4D
Ssc5D: soluble scavenger with five domains
T2DM: type 2 diabetes mellitus
THBS1: thombospondin‐1
TIMP‐1: tissue inhibitor of metalloproteinase
TLM3: TNO‐LightGBM Model consisting of three biomarkers
TNC: tenascin C
VCAN: versican
VCTE: vibration‐controlled transient elastography
WHR: waist‐to‐hip ratio

**Summary**

- Metabolic dysfunction‐associated steatotic liver disease (MASLD) fibrosis can lead to serious health problems or even death, often without noticeable symptoms until advanced stages of disease occur.
- This study found that certain proteins in the blood are linked to early signs of liver damage in both mice and humans.
- Detection of these proteins may detect early onset of MASLD‐fibrosis and may thus help better manage treatment for patients at risk of MASLD.

## 1 Introduction

Metabolic dysfunction‐associated steatotic liver disease (MASLD), and its progressive form metabolic dysfunction‐associated steatohepatitis (MASH), is the most prevalent chronic liver disease, of high significance to hepatology and to clinical specialties concerned with insulin resistance and cardiometabolic risk such as diabetology and preventive cardiology [1, 2]. Globally, the prevalence of MASLD lies around 30% while patients with type 2 diabetes mellitus (T2DM) have very high rates of around 60%–70% [3, 4]. The prevalence of advanced liver fibrosis is suggested to be around 3% in the general population [5, 6] but increases to 17% in patients with T2DM [7].

Liver fibrosis progression is highly variable in the MASLD population. Some patients with MASLD develop advanced liver fibrosis or even cirrhosis within a relatively short time span, while others remain stable or even regress. A systematic review and meta‐analysis of paired liver biopsy studies demonstrated that approximately 33% of patients with MASLD developed fibrosis progression during follow‐up, while 43% had no significant change in fibrosis stage, negating the notion that fibrosis in MASLD is a one‐way progression towards worsening disease [8]. Similarly, these studies reported that about 37% of patients with MASH progressed to fibrosis over an average follow‐up period of 5–7 years, highlighting the variable trajectory of fibrosis in these patients [8, 9]. These findings underscore the need for a better understanding of fibrosis dynamics in MASLD, including both progressive and stable disease trajectories. To map these disease trajectories, blood‐based biomarkers linked to disease progression could be preferred over more invasive repeated liver biopsies, allowing for early identification of patients with MASLD at risk for disease progression.

Over the last decades, several non‐invasive tests (NITs) have been developed to distinguish stages of MASLD. Two widely used tests are the enhanced liver fibrosis test (ELF) and vibration‐controlled transient elastography (VCTE) by FibroScan. ELF uses three distinct biomarkers of fibrogenesis to determine the presence of liver fibrosis, namely hyaluronic acid (HA), procollagen III amino terminal peptide (PIIINP) and tissue inhibitor of metalloproteinase 1 (TIMP‐1) [10]. VCTE measures the attenuation of the ultrasound signal to derive the controlled attenuation parameter (CAP), which is used as a proxy for steatosis, and measures the speed of a shear wave across the liver to derive a liver stiffness measurement (LSM), a marker for liver fibrosis.

However, even though these NITs are suited to detect the presence of fibrotic MASLD, they are unable to prospectively identify patients at risk of developing fibrosis in the future. Since MASLD often remains unnoticed until advanced stages of disease, it is highly relevant to develop means to predict disease progression. Even before the development of cirrhosis, patients with (fibrotic) MASLD are at increased risk of overall and liver‐related mortality and increased likelihood of liver‐related complications such as hepatocellular carcinoma [11, 12]. Accurate prediction of which patients are likely to develop severe stages of disease would allow early initiation of treatment and targeted, effective reassessments.

Recently, we developed a novel biomarker panel (TLM3), consisting of semaphorin‐4D (Sema4D), soluble scavenger with five domains (Ssc5D) and insulin‐like growth factor‐binding protein 7 (IGFBP7), that demonstrated high accuracy in distinguishing histological MASLD fibrosis stages (F0/1: area under the curve (AUC) 0.82; F2: AUC 0.89; F3/4: AUC 0.87) using data and samples originating from multiple cross‐sectional human studies [13]. TLM3 was derived from a larger fibrosis biomarker panel, which included TNC, FBN1, Sema4D, ADAMTS, PAM, Ssc5D, VCAN, Urokinase, THBS1, IGFBP7 and CXCL10, and was identified by associating serum protein levels to active disease processes, as discovered in a pre‐clinical study [13]. We hypothesise that circulating concentrations of these biomarkers may serve as a predictor for fibrosis development at follow‐up. We first tested this hypothesis by analysing available data from two time‐course studies using a diet‐induced LDLr^−/−^.Leiden MASLD mouse model [14] and a diet‐induced obesity mouse model [15]. Subsequently, we used samples available from the longitudinal prospective multi‐ethnic general population HELIUS cohort [16, 17] to correlate the serum levels of the fibrosis biomarker panel at baseline with established liver fibrosis proxies, i.e., FIB4, VCTE and ELF, at on average 7‐year follow‐up, and to determine the diagnostic accuracy, defined as the AUC, of the fibrosis biomarker panel for new onset liver fibrosis, defined as ELF ≥ 9.8 at follow‐up in participants compared to ELF < 9.8 at baseline.

## 2 Participants and Methods

### 2.1 Animal Study

This study was approved by an independent Animal Care and Use Committee and was in compliance with European Community specifications for the use of laboratory animals. Briefly, 12‐week‐old male LDLr^−/−^.Leiden mice received normal chow (*n* = 6 per timepoint) or a high‐fat diet (*n* = 12 per timepoint) and were sacrificed at 0, 6, 12, 18 and 24 weeks after initiating the diets. Liver tissue was partly fixated in formalin and paraffin‐embedded for histological examination and partly snap frozen in liquid nitrogen for RNA isolation. Liver fibrosis was assessed histochemically by Picro‐Sirius Red staining (Chroma; WALDECK‐GmbH, Munster, Germany). The development of fibrosis (typically first apparent in the mouse model after 18 weeks on high‐fat diet [14]) was assessed by a liver pathologist to quantify the percentage of perisinusoidal fibrosis (expressed as the percentage of perisinusoidal fibrosis relative to the total perisinusoidal area). Liver gene expression was measured by RNA‐sequencing performed by BaseClear BV (Leiden, The Netherlands). The libraries were multiplexed, clustered and sequenced on an Illumina HiSeq 2500 with a single‐read 50‐cycle sequencing protocol, 15 million reads per sample and indexing. The bioconductor DESeq2 package was used to compare gene expression profiles to chow control mice at each timepoint and generate fold change and *p*_adj_‐values. *p*_adj_‐values < 0.01 were considered to be significantly regulated. These data originate from a previously published time‐course study. A detailed description of the in vivo study and subsequent measurements are reported elsewhere [14]. Additionally, results were validated using liver gene expression data of a time‐resolved study in diet‐induced ob/ob mice receiving either chow (*n* = 5) or Fast Food diet (*n* = 5) sacrificed at 0, 2, 4, 8, 12, 18, 24 and 30 weeks after initiating the diet. A detailed description of this in vivo study and subsequent measurements are reported elsewhere [15].

### 2.2 Study Population

This study uses data and biobank material of the multi‐ethnic healthy life in an urban setting (HELIUS) study and the NAFLD in the healthy life in an urban setting (NILE) study, which is a sub‐study of the HELIUS study. HELIUS is a population‐based prospective cohort study at the Amsterdam University Medical Center (Amsterdam UMC) in Amsterdam, The Netherlands, and has previously been described [16, 17, 18]. The HELIUS study includes participants from six ethnic groups (of Dutch, African Surinamese, South‐Asian Surinamese, Moroccan and Ghanaian origin) and aims to gain insight into the impact of ethnicity on health status in adults living in Amsterdam, The Netherlands. A total of 24 780 participants attended a baseline visit between 2011 and 2015. Of those, 10 585 had a follow‐up visit between 2019 and 2021, of whom a selection of 399 participants attended the NILE VCTE sub‐study approximately 7 years after the baseline HELIUS study visit [17]. This selection consisted of participants at risk of MASLD (*n* = 346 participants) and a control group (*n* = 53 participants). Exclusion criteria were, among others, excessive alcohol intake, a known histology of viral hepatitis and use of systemic corticosteroids [17].

Data on demographic variables (age, sex, ethnic background), laboratory results (including aspartate transaminase [AST], alanine transaminase [ALT], cholesterol, glucose and HbA1c), cardiovascular risk factors (including body mass index [BMI], waist‐to‐hip ratio [WHR] and presence of T2DM), and self‐reported alcohol use were collected from the municipal registry or during baseline and follow‐up visits. T2DM was determined based on self‐report, a single measurement of fasting glucose ≥ 7.0 mmol/L and/or the use of glucose‐lowering medication. Moreover, during the follow‐up visit, as part of the NILE study, platelets were measured and VCTE, consisting of CAP as a proxy for steatosis and LSM as a proxy for fibrosis, were performed. VCTE was performed after a fast of at least 4 h. All VCTE measurements were performed by the same highly experienced physician (A.v.D.).

A total of 80 participants were included in the current study, divided into 4 groups based on the following criteria: 20 participants with metabolic risk factors (defined as T2DM and/or overweight [BMI ≥ 25 kg/m^2^]) at baseline and LSM ≥ 9.0 kPa at follow‐up; 20 participants with metabolic risk factors at baseline and LSM 7.0–9.0 kPa at follow‐up; 20 participants with metabolic risk factors at baseline and LSM < 7.0 kPa at follow‐up; and 20 participants without metabolic risk factors at baseline and LSM < 7.0 kPa at follow‐up. This selection was made to ensure that the study population included participants who experience disease progression during the follow‐up period. Figure 2 shows the flow of inclusions.

### 2.3 Sample Collection and Storage

Blood samples of the HELIUS and NILE study were collected as previously published [16, 17]. Following centrifugation and serum isolation, samples were stored in the designated biobank at the Amsterdam UMC at −80°C until they were used for analyses. As such, all samples had undergone one freeze–thaw cycle.

### 2.4 ELF and Fibrosis Biomarker Panel Analyses

ELF was calculated based on the measurements of three biomarkers of fibrogenesis, namely HA, PIIINP and TIMP‐1, performed at the endocrinology laboratory at the Amsterdam UMC. The three different ELF proteins were analysed separately using ELF kits according to manufacturers' instructions using the Atellica IM analyser (Siemens Heathineers) [19]. For interpretation of ELF, a cut‐off value of > 9.8 was used to identify patients at increased risk of advanced fibrosis (≥ F3) [19, 20].

The fibrosis biomarker panel identified in the translational animal model was measured in human participants. Assays were performed according to the manufacturer's instructions as previously described [13].

### 2.5 Data Analyses

Statistical analyses were conducted in R (version 4.3.2). *p*‐values < 0.05 were considered significant. Participant characteristics are expressed as frequencies with percentages, means with standard deviations (±SD) or medians with interquartile range (IQR), depending on data distribution, and are given at baseline and follow‐up. Pearson's or Spearman's *R* correlation was calculated between the fibrosis biomarker panel at baseline and FIB4, VCTE and ELF at follow‐up. Additionally, linear regression between fibrosis biomarkers at baseline and FIB4, VCTE and ELF at follow‐up was calculated.

To explore the ability of the fibrosis biomarker panel at baseline to predict ≥F3 fibrosis at follow‐up, participants with ELF ≥ 9.8 at baseline were omitted from analyses. The remaining study population was dichotomised for ELF at follow‐up. Participant characteristics stratified for ELF at follow‐up are presented. Boxplots were utilised to visualise the spread of the fibrosis biomarker panel within the two ELF groups and are accompanied by Mann–Whitney *U* or unpaired *t*‐test, depending on the data distribution. AUC of the fibrosis biomarkers at baseline to detect ELF ≥ 9.8 was calculated and visualised using receiver operating characteristic (ROC) curves.

## 3 Results

### 3.1 Preclinical Model

**FIGURE 1:**
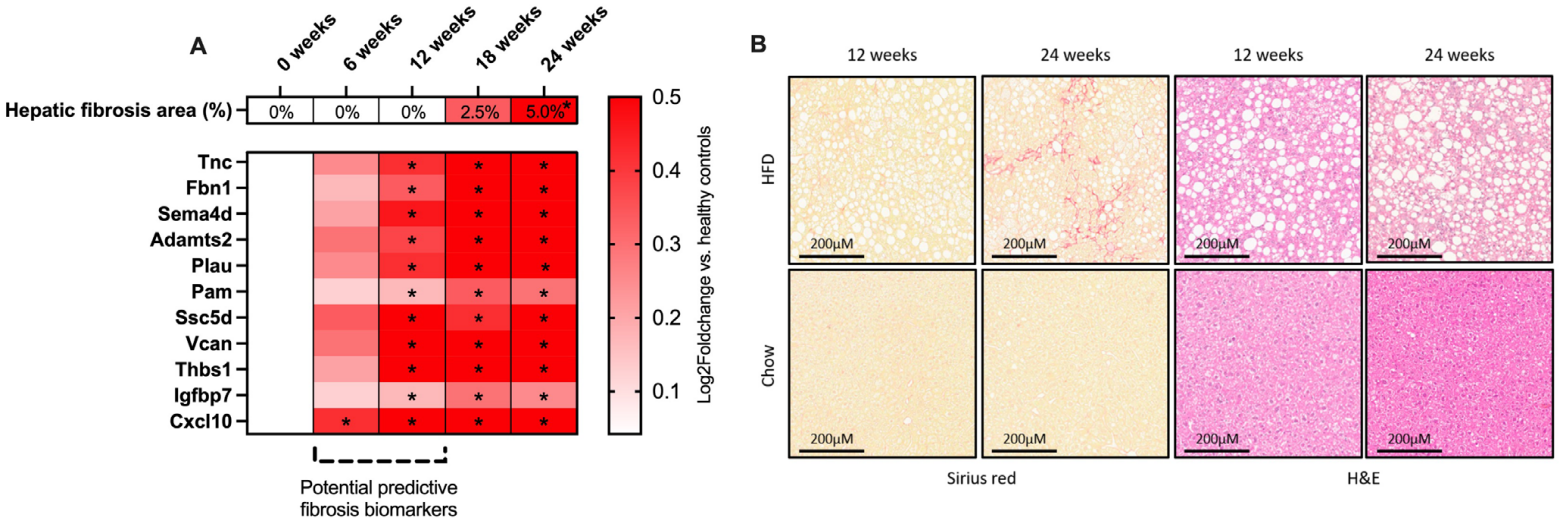
Preclinical mouse model. Histochemically obtained data on hepatic fibrosis and gene expression of genes encoding the proteins in the fibrosis biomarker panel (A) and Sirius Red staining and H&E staining of HDF and Chow‐diet mouse at 12‐ and 24‐weeks (B). *p < 0.05. H&E = haematoxylin and eosin; HFD = high‐fat‐diet.

To assess the potential predictive capacity of the novel fibrosis biomarker panel [13], we investigated whether the expression of the genes encoding the fibrosis signature is upregulated before hepatic fibrosis becomes apparent. This change in gene expression was determined in a longitudinal study using a high‐fat diet‐induced MASLD mouse model (LDLr−/−.Leiden mice). Significant hepatic fibrosis was first detected on a histological level at 24 weeks, while the expression of the genes encoding the fibrosis biomarker panel was upregulated earlier (Figure 1A). At 6 weeks, the expression of *Cxcl10* was already significantly increased compared to chow control mice, and at 12 weeks the expression of *Sema4d*, *Vcan*, *Plau*, *Pam*, *Fbn1*, *Tnc*, *Cxcl10*, *Ssc5d*, *Igfbp7*, *Adamts2* and *Thbs1* was significantly increased (Figure 1A). Figure 1B shows Sirius Red staining and haematoxylin and eosin (H&E) staining of HFD mice compared to chow control mice at 12 and 24 weeks. Additionally, the FFD diet‐induced obesity model supports these findings and demonstrates that hepatic fibrosis is notably induced after 8 weeks of FFD feeding, as indicated by an increase in collagen deposition at this time point (Figure S1). Consistent with earlier observations in the MASLD model, gene expression analysis demonstrated that pro‐fibrogenic genes are regulated early, with some upregulated as early as 2 weeks and all fibrogenic genes being regulated by 4 weeks of FFD feeding compared to chow (Figure S1). This suggests pre‐symptomatic gene regulation before pathology becomes apparent. As previously validated, these genes encode proteins that can be measured in human circulation [13].

### 3.2 Participant Characteristics

**FIGURE 2:**
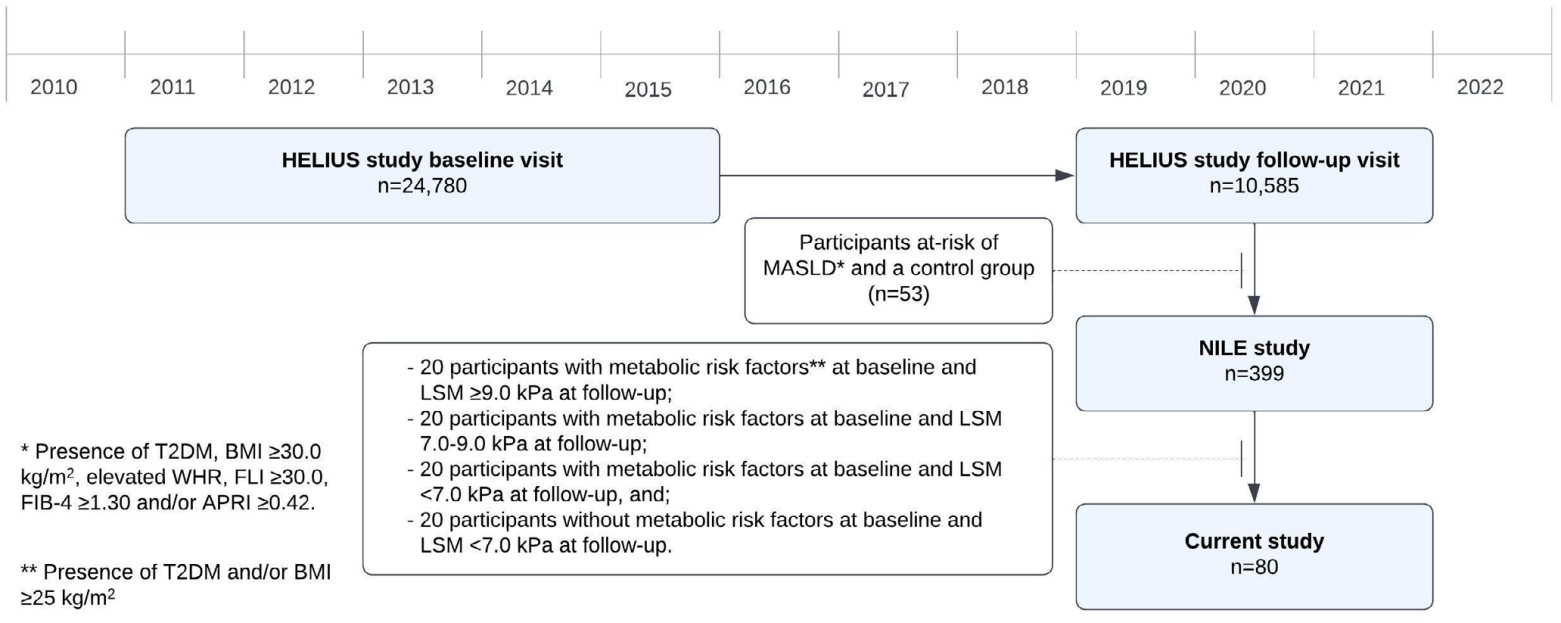
Flowchart of inclusions. APRI = AST to platelet ratio; BMI = body mass index; FIB4 = fibrosis‐4 score; LSM = liver stiffness measurement; MASLD = metabolic dysfunction‐associated steatotic liver disease; T2DM = type 2 diabetes mellitus; WHR = waist‐to‐hip circumference.

Subsequently, we measured the concentration of these proteins in blood samples collected from participants at risk of MASLD in longitudinal studies. Table 1 shows participant characteristics of the entire study population at baseline and at follow‐up, and Figure 2 shows the flow of inclusions. Median follow‐up duration was 7.4 (IQR: 6.3–7.9) years. 52.5% of participants were women, and mean age at baseline was 50.2 (±11.5) years. Mean ELF increased from 8.7 (±0.8) at baseline to 9.3 (±0.9) (*p* < 0.001) at follow‐up. At baseline, 10 participants (12.5%) had an ELF ≥ 9.8, compared to 17 participants (21.5%) at follow‐up. 81.2% of participants had CAP ≥ 238 dB/m, and 30.1% had LSM ≥ 8.2 kPa at follow‐up.

**TABLE 1:** Participants' characteristics at baseline and follow‐up visits.

		Baseline (*n* = 80 participants)	Follow‐up (*n* = 80 participants)	*p*
Age, years (mean [SD])		50.2 (11.5)	57.3 (11.6)	< 0.001*
Sex, *n* women (%)		42 (52.5)		NA
Ethnic origin	Dutch, *n* (%)	40 (50.0)		NA
	South Asian Surinamese, *n* (%)	28 (35.0)		
	African Surinamese, *n* (%)	8 (10.0)		
	Ghanaian, *n* (%)	2 (2.5)		
	Moroccan, *n* (5)	2 (2.5)		
T2DM, *n* (%)		17 (21.2)	22 (27.5)	0.461**
BMI, kg/m^2^ (mean [SD])		29.8 (6.0)	30.4 (6.3)	0.572*
Waist circumference, cm (mean [SD])		NA	103.8 (16.1)	NA
Laboratory measurements	Glucose, mmol/L (median [IQR])	5.6 (5.0, 6.1)	6.0 (5.3, 6.8)	0.014***
	HbA1c, mmol/mol (median [IQR])	39 (36, 43)	39 (35, 48)	0.521***
	Total cholesterol, mmol/L (mean [SD])	5.04 (1.14)	5.05 (1.22)	0.933*
	LDL, mmol/L (mean [SD])	3.05 (1.06)	3.02 (1.11)	0.835*
	HDL, mmol/L (mean [SD])	1.31 (0.42)	1.41 (0.39)	0.117*
	Triglycerides, mmol/L (median [IQR])	1.12 (0.72, 1.82)	1.14 (0.86, 1.73)	0.624***
	Platelets, ×10^9^ (median [IQR])	NA	246 (191, 298)	NA
FIB4 (median [IQR])		NA	1.32 (0.88, 1.63)	NA
FIB4 categorical	< 1.30, *n* (%)	NA	36 (45.0)	NA
	1.30–2.67, *n* (%)	NA	35 (43.8)	NA
	2.67–3.25, *n* (%)	NA	5 (6.2)	NA
	≥ 3.25, *n* (%)	NA	3 (3.8)	NA
ELF (mean [SD])		8.74 (0.82)	9.31 (0.94)	< 0.001*
ELF categorical	< 9.8, *n* (%)	70 (87.5)	62 (78.5)	0.144**
	≥ 9.8, *n* (%)	10 (12.5)	17 (21.5)	
CAP, dB/m (median [IQR])		NA	310 (256, 353)	NA
CAP categorical	< 238 dB/m, *n* (%)	NA	15 (18.8)	NA
	238–260 dB/m, *n* (%)	NA	7 (8.8)	NA
	260–290 dB/m, *n* (%)	NA	13 (16.2)	NA
	≥ 290 dB/m, *n* (%)	NA	45 (56.2)	NA
LSM, kPa (median [IQR])		NA	7.05 (4.45, 8.82)	NA
LSM categorical	< 8.2 kPa, *n* (%)	NA	56 (70.0)	NA
	8.2–9.7 kPa, *n* (%)	NA	7 (8.8)	NA
	9.7–13.6 kPa, *n* (%)	NA	15 (18.8)	NA
	≥ 13.6 kPa, *n* (%)	NA	2 (2.5)	NA

Abbreviations: BMI = body mass index; CAP = controlled attenuation parameter; ELF = enhanced liver fibrosis‐score; FIB4 = fibrosis‐4 score; HbA1c = haemoglobulin A1c; HDL = high‐density lipoprotein; IQR = interquartile range; LDL = low‐density lipoprotein; LSM = liver stiffness measurement; SD = standard deviation; T2DM = type 2 diabetes mellitus.

* Unpaired *t*‐test.

** Chi‐square test.

*** Mann–Whitney *U*‐test.

### 3.3 Correlation With Fibrosis Proxies

**FIGURE 3:**
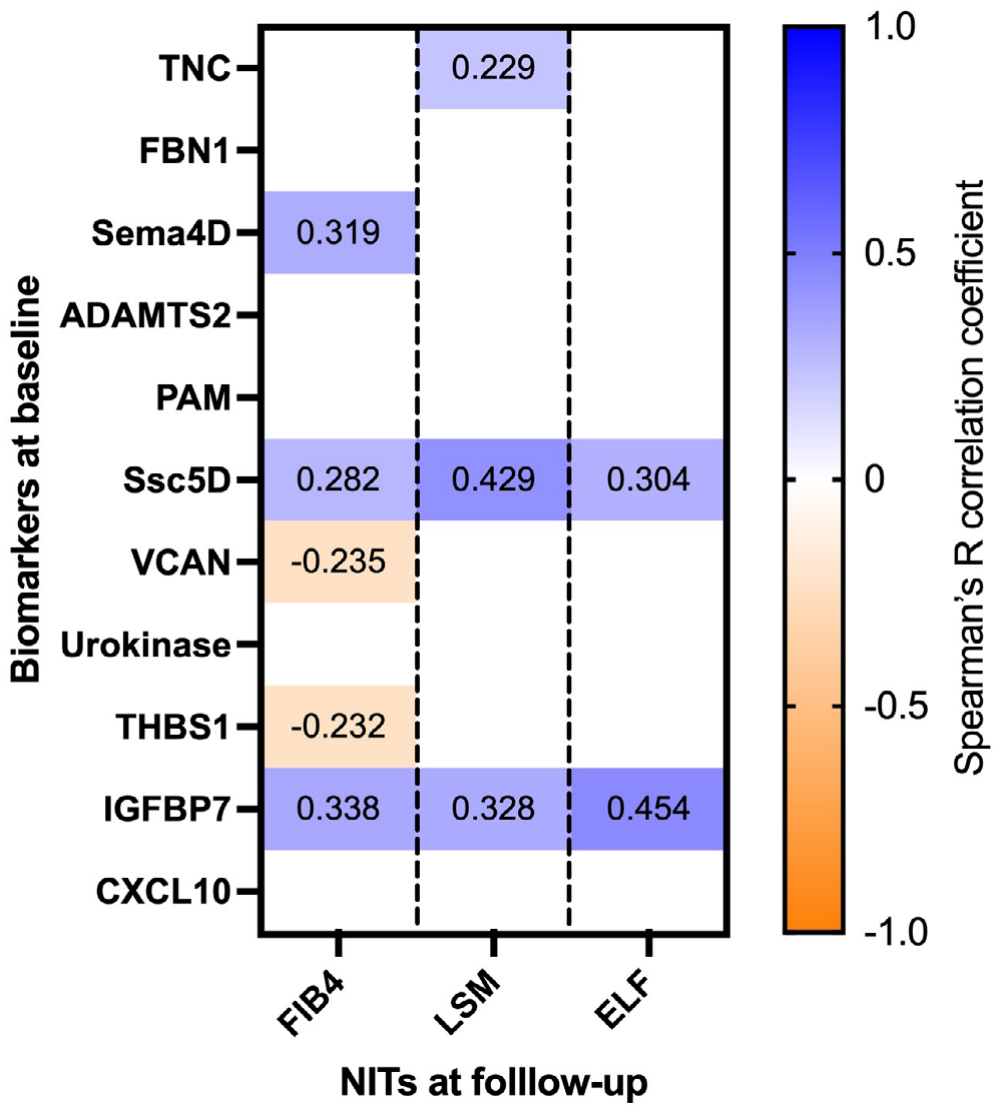
Correlogram. Correlogram showing significant Spearman's *R* correlation between fibrosis biomarkers at baseline and established liver NITs (FIB4, LSM and ELF) at follow‐up. ELF = enhanced liver fibrosis score; FIB4 = fibrosis‐4 score; LSM = liver stiffness measurement.

Spearman's *R* correlation coefficients were calculated to investigate correlations between the fibrosis biomarker panel concentrations at baseline and established liver fibrosis NITs, i.e., FIB4, LSM and ELF, at follow‐up (median 7.4 years) (Figure 3, Table S1). With FIB4 as a reference for fibrosis, significant correlations were found for ‐Sema4D (*R* = 0.21, *p* = 0.004), Ssc5D (*R* = 0.282, *p* = 0.012), versican (VCAN) (*R* = −0.235, *p* = 0.037), thrombospondin‐1 (THBS1) (*R* = −0.232, *p* = 0.040) and IGFBP7 (*R* = 0.34, *p* = 0.002). With LSM as a reference for fibrosis at follow‐up, significant correlations were found for tenascin C (TNC) (*R* = 0.229, *p* = 0.041), Ssc5D (*R* = 0.43, *p* < 0.001) and IGFBP7 (*R* = 0.33, *p* = 0.003). Lastly, when using ELF as a reference for fibrosis at follow‐up, significant correlations were found for Ssc5D (*R* = 0.30, *p* = 0.007) and IGFBP7 (*R* = 0.45, *p* = 0.026) again.

### 3.4 Diagnostic Accuracy for New Onset Fibrosis

To explore the ability of the fibrosis biomarkers at baseline to predict the incidence of ≥ F3 fibrosis at follow‐up, participants with ELF < 9.8 at baseline (*n* = 70) were used for analyses. At follow‐up, 57 out of 70 (81.4%) participants still had ELF < 9.8 and 13 (18.6%) had developed ELF ≥ 9.8 (Table S2). Participants with ELF < 9.8 at follow‐up were younger (mean age at baseline: 47.3 [±11.0] years) than participants who had ELF ≥ 9.8 at follow‐up (55.2 [±10.3] years [*p* = 0.019]). Participants with ELF < 9.8 at follow‐up also had lower median FIB4 at follow‐up (1.2 [IQR: 0.9–1.5]) compared to those with ELF ≥ 9.8 at follow‐up (2.0 [IQR: 1.3–3.0] [*p* = 0.010]). Of note, these groups did not differ regarding the presence of T2DM, mean BMI, platelet count or markers of glucose and lipid metabolism at baseline or at follow‐up.

**FIGURE 4:**
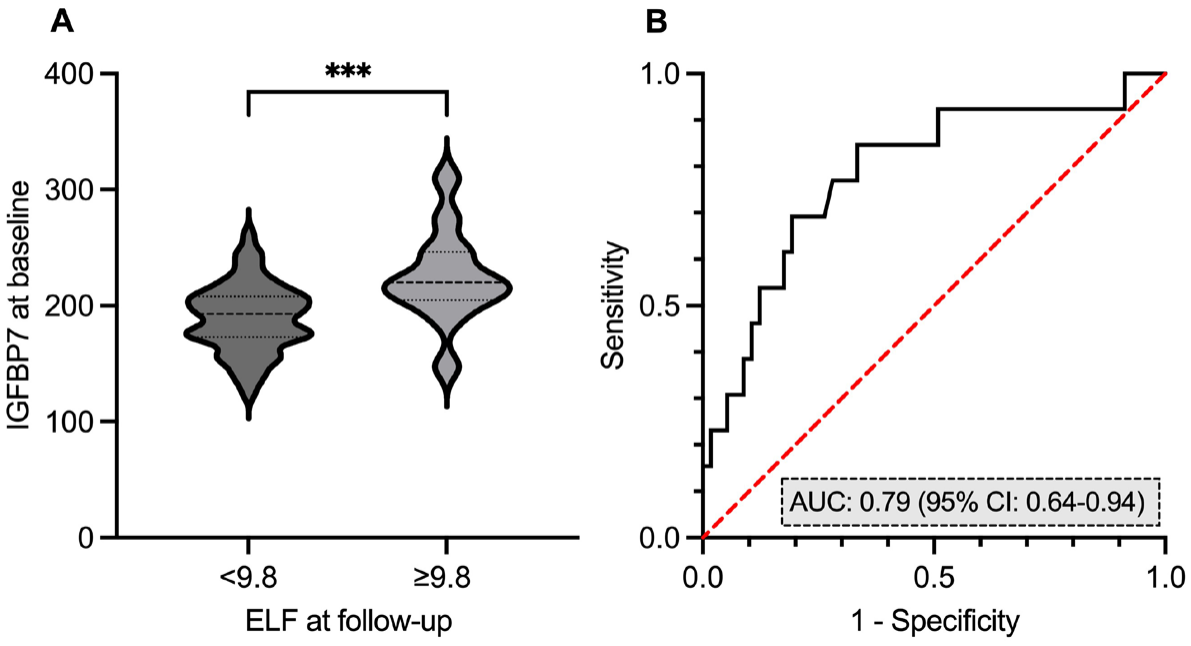
IGFBP7 for the detection of new onset fibrosis. Boxplot of the concentration of IGFBP7 at baseline stratified for ELF at follow up in participants with ELF < 9.8 at baseline (A) and ROC curve of IGFBP7 for the detection of ELF ≥ 9.8 in participants with ELF < 9.8 at baseline (B) (*n* = 70 participants). ***p < 0.005. ELF = enhanced liver fibrosis score; ROC curve = receiver operating characteristic curve.

Median IGFBP7 at baseline was higher in the group who developed liver fibrosis according to ELF ≥ 9.8 at follow‐up compared to the group with ELF remaining < 9.8 at follow‐up (220.0 [IQR: 207.6–243.6] vs. 192.8 [IQR: 173.3–207.9] [*p* = 0.001]) (Figure 4A, Table S3). Other fibrosis biomarkers did not differ between the two groups. IGFBP7 had an AUC of 0.79 (95% CI: 0.64–0.94) for the detection of ELF ≥ 9.8 at follow‐up (Figure 4B, Table S4). After adjustment for age, IGFBP7 remained a significant predictor in a multivariate logistic regression model. Other biomarkers did not yield significant results.

## 4 Discussion

Here, we showed that a previously published TLM3 fibrosis biomarker panel [13] has predictive capabilities on a gene level in two different murine models, viz. the LDLr−/−.Leiden MASLD model and the obesity (ob/ob) model. In addition, the fibrosis biomarkers in serum correlate at baseline with established NITs for liver fibrosis at a median follow‐up of 7 years, and components of TLM3 can predict new onset fibrosis with a good degree of accuracy in the general population at risk for MASLD. First, we observed that genes encoding the fibrosis biomarker panel were upregulated prior to the onset of histopathological features of liver fibrosis in a translational mouse model for human MASLD. Translating to human in a second step, with a selection of participants at risk for MASLD from a longitudinal general population study with a median follow‐up of 7 years, we found that baseline serum concentrations of five of these markers, being Sema4D, Ssc5D, VCAN, THBS1 and IGFBP7 correlated with the liver fibrosis proxy FIB4 at follow‐up; that three of these markers, being TNC, Ssc5D and IGFBP7 correlated to LSM at follow‐up, and that two of these markers at baseline, being Ssc5D and IGFBP7 correlated to ELF at follow‐up. Moreover, we showed that IGFBP7 serum levels at baseline can predict new onset liver fibrosis over time, defined as ELF ≥ 9.8 at follow‐up in participants with ELF < 9.8 at baseline, with good diagnostic accuracy (AUC 0.79). Together, these murine and human analyses raise the hypothesis that the mouse‐derived fibrosis biomarker for MASLD‐fibrosis may capture an early stage of the disease and even predict the occurrence of actual fibrosis.

Among the tested 11 biomarkers at baseline, Ssc5D and IGFBP7 are particularly noteworthy as they showed the highest correlation coefficients with NITs at follow‐up and their concentrations at baseline were significantly correlated with FIB4, LSM and ELF at follow‐up, therefore increasing the likelihood that they are reliable predictive markers for liver fibrosis development. Interestingly, we recently demonstrated the utility of these biomarkers in cross‐sectional cohorts where they, as part of TLM3, effectively differentiated between stages of MASLD‐fibrosis [13]. Functionally, IGFBP7 contributes to fibrogenesis by playing a role in the activation and transdifferentiation of hepatic stellate cells [21], and knockdown of IGFBP7 in mouse models of MASLD resulted in reduced insulin resistance and hepatic steatosis [22]. Ssc5D is a soluble receptor expressed on macrophages and T cells [23].

The selection of participants in this study was based on cardiometabolic risk factors at baseline and VCTE data at follow‐up. We chose this design to enhance the likelihood of participants in the study cohort developing fibrosis over time, as the prevalence of advanced MASLD‐fibrosis remains relatively low in general population cohorts, even with cardiometabolic risk factors. A limitation of the current study is the unavailability of liver histology as a reference standard. We utilised multiple NITs at baseline and at follow‐up as proxies for fibrosis. With these established proxies, we delineated the development of fibrosis within the study cohort. The ability of NITs in diagnosing MASLD and distinguishing stages of MASLD has increasingly been established [24]. Moreover, NITs, including FIB4, ELF and VCTE are associated with liver‐related outcomes [25, 26, 27], and simple NITs may perform as well as histologically assessed fibrosis in predicting clinical outcomes in patients with MASLD [28]. ELF has been shown to increase with age [29], but the effect size for the prediction of fibrosis development is too large to be explained by age alone. Moreover, after adjustment for age, IGFBP7 remained significant in a multivariate logistic regression model.

Our study presents unique murine discovery data and also unique longitudinal data of participants from a general population cohort at cardiometabolic risk of MASLD. For future studies, a bigger sample size and longer follow‐up duration would allow inclusion of relevant clinical outcomes, i.e., liver cirrhosis, hepatocellular carcinoma and mortality. Moreover, the combination of NITs and more invasive and/or costly diagnostic tools such as multiparametric MRI and liver biopsy can increase validation of the prognostic markers in future studies.

Our findings also highlight the added value of employing a translational animal model for biomarker research. The fibrosis biomarker panel used in this study was originally found through studying the dynamics of transcriptional changes associated with hepatic collagen/extracellular matrix synthesis in the diet‐induced MASLD LDLr^−/−^.Leiden mouse model. This is a well‐characterised mouse model, often used in pre‐clinical MASLD research due to its profound translational value [30, 31]. Such mouse models allow for the integration of tracer‐ and omics technologies beyond what can be investigated in the human setting. This combination facilitates the identification of biomarkers strongly associated with the dynamics of disease mechanisms. Importantly, as shown in the current publication, such an approach even allows for the identification of predictive biomarkers for disease onset, reinforcing the unique potential of translational animal models for biomarker discovery. Due to the unique approach of coupling the dynamics of disease mechanism with pathology, we were able to identify a set of biomarkers that was able to predict fibrosis 7 years before symptoms emerged, allowing for improved risk stratification of patients at‐risk of MASLD even before the onset of fibrosis, thus hopefully improving treatment allocation and preventing disease progression and adverse disease outcomes.

## Author Contributions

Conceptualisation: K.C.S., J.C.B.C.J., A.‐M.D., M.E.T., R.H., L.V., A.G.H. Methodology: K.C.S., J.C.B.C.J., S.Ö., J.S., A.K., A.‐M.D., L.V., A.G.H. Software: not applicable. Validation: K.C.S., J.C.B.C.J., M.P.M.C., Q.J.J.A., L.V., A.G.H. Formal analysis: K.C.S., J.C.B.C.J., S.Ö. Investigation: K.C.S., J.C.B.C.J., S.Ö., M.P.M.C., Q.J.J.A., J.S., A.K., H.M.H., L.V., A.G.H. Resources: J.S., A.K., H.G., A.‐M.D., B.‐J.B., L.L.G., L.V., A.G.H. Data curation: K.C.S., J.C.B.C.J., J.S., A.K., H.G., A.‐M.D., A.L.M., L.V., A.G.H. Writing – original draft: K.C.S., J.C.B.C.J., L.V. Writing – review and editing: K.C.S., J.C.B.C.J., S.Ö., M.P.M.C., Q.J.J.A., J.S., A.K., H.G., H.M.H., A.‐M.D., A.L.M., B.‐J.B., M.N., J.P.H.D., L.L.G., M.E.T., R.H., L.V., A.G.H. Visualisation: K.C.S., J.C.B.C.J. Supervision: J.P.H.D., M.E.T., R.H., L.V., A.G.H. Project administration: K.C.S., H.G., A.‐M.D., L.V., A.G.H. Funding acquisition: H.G., B.‐J.B., M.N., J.P.H.D., L.L.G., M.E.T., R.H., A.G.H.

## Ethics Statement

This study was approved by the Medical Ethics Committee (METC) of the Amsterdam UMC, and conducted in accordance with the Declaration of Helsinki. Written informed consent was obtained from all human participants, and all animal procedures were performed in compliance with institutional guidelines for the care and use of animals.

## Consent

Informed consent was obtained from all participants involved in this study. Participants were provided with detailed information regarding the study's purpose, procedures and potential risks, and they gave their written consent to participate. Confidentiality of all personal data was maintained in accordance with ethical guidelines and privacy regulations.

## Conflicts of Interest

M.N. is on the Scientific Advisory Board of Caelus Pharmaceuticals and Advanced Microbiome Interventions, the Netherlands. However, none of these bear direct relevance to the current manuscript. Other authors have no conflicts of interest.

## Supporting information


**Figure S1.** Validation FFD‐diet‐induced obesity model. Percentage of hepatic fibrosis based on histological analysis of Picro Sirius Red staining of ob/ob mice fed normal control diet (NCD), choline‐deficient, L‐amino acid‐defined, high‐fat diet (CDAHFD) or fast food diet (FFD) (A). Gene expression of genes after 2 and 4 weeks of FFD feeding in ob/ob mice (B). Significant gene expression is indicated by bold *p*‐values.

Data S1.

## Data Availability

The data that support the findings of this study are available from the corresponding author upon reasonable request. The datasets generated and/or analysed during the current study are not publicly available due to privacy and ethical considerations but may be shared upon request for non‐commercial research purposes.
