# Supplementary figures and images for: Prognostic Value of the TLM3 Biomarker Panel for Early Fibrosis Development in MASLD Within the General Population
Source: Liver Int. 2025 Jun 24;45(7):e70169. doi: 10.1111/liv.70169 (PMC12186288; doi:10.1111/liv.70169)

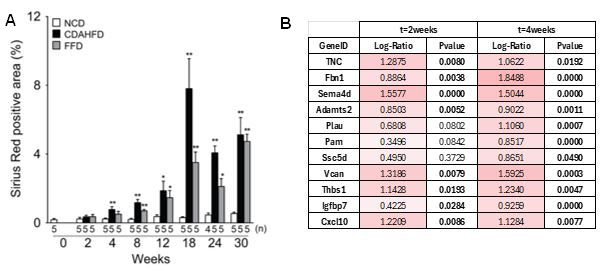

Supplement: Supplementary file 1 — Figure S1. Validation FFD‐diet‐induced obesity model. Percentage of hepatic fibrosis based on histological analysis of Picro Sirius Red staining of ob/ob mice fed normal control diet (NCD), choline‐deficient, L‐amino acid‐defined, high‐fat diet (CDAHFD) or fast food diet (FFD) (A). Gene expression of genes after 2 and 4 weeks of FFD feeding in ob/ob mice (B). Significant gene expression is indicated by bold p‐values. [file LIV-45-0-s002.jpg]
