# Supplementary material for: Prognostic Value of the TLM3 Biomarker Panel for Early Fibrosis Development in MASLD Within the General Population
Source: Liver Int. 2025 Jun 24;45(7):e70169. doi: 10.1111/liv.70169 (PMC12186288; doi:10.1111/liv.70169)
Supplement: Supplementary file 2 — Data S1. [file LIV-45-0-s001.docx]

Supplementary tables

|  |  | NITs |  |  |  |  |  |
| --- | --- | --- | --- | --- | --- | --- | --- |
|  |  | FIB4 |  | LSM |  | ELF |  |
|  |  | Correlation coefficient | p-value | Correlation coefficient | p-value | Correlation coefficient | p-value |
| Biomarkers at baseline | TNC | -0.189 | 0.096 ^C^ | 0.229 | 0.041 ^C^ | 0.134 | 0.238 ^C^ |
|  | FBN1 | 0.001 | 0.963 ^C^ | -0.038 | 0.740 ^C^ | 0.009 | 0.940 ^C^ |
|  | Sema4D | 0.319 | 0.004 ^C^ | 0.099 | 0.383 ^C^ | 0.084 | 0.463 ^C^ |
|  | ADAMTS | 0.042 | 0.712 ^C^ | -0.083 | 0.464 ^C^ | 0.032 | 0.780 ^C^ |
|  | PAM | -0.101 | 0.378 ^C^ | 0.067 | 0.553 ^C^ | -0.067 | 0.558 ^C^ |
|  | Ssc5D | 0.282 | 0.012 ^C^ | 0.429 | <0.001 ^C^ | 0.304 | 0.007 ^C^ |
|  | VCAN | -0.235 | 0.037 ^C^ | -0.001 | 0.991 ^C^ | 0.029 | 0.799 ^C^ |
|  | Urokinase | 0.217 | 0.054 ^C^ | -0.034 | 0.767 ^C^ | 0.154 | 0.176 ^C^ |
|  | THBS1 | -0.232 | 0.040 ^C^ | -0.093 | 0.410 ^C^ | -0.065 | 0.569 ^C^ |
|  | IGFBP7 | 0.338 | 0.002 ^C^ | 0.328 | 0.003 ^C^ | 0.454 | 0.026 ^C^ |
|  | CXCL10 | -0.098 | 0.390 ^C^ | -0.015 | 0.897 ^C^ | 0.115 | 0.311 ^C^ |

**Supplementary table 1: Correlation of fibrosis biomarkers at baseline and established liver NITs at follow-up**. Spearman’s R correlation coefficients of biomarkers at baseline and FIB4, LSM and ELF at follow-up (n=80 participants). NITs = non-invasive tests; FIB4 = fibrosis-4 score; LSM = liver stiffness measurement; ELF = enhanced liver fibrosis-score. ^C^ = Mann-Whitney U-test.

|  |  | ELF at follow-up <9.8 (n = 57) | ELF at follow-up ≥9.8 (n = 13) | p-value |
| --- | --- | --- | --- | --- |
| Sex, n women (%) |  | 32 (56.1) | 6 (46.2) | 0.552^B^ |
| Ethnic origin | Dutch, n (%) | 32 (56.1) | 4 (30.8) | 0.317^B^ |
|  | South Asian Surinamese, n (%) | 19 (33.3) | 7 (53.8) |  |
|  | African Surinamese, n (%) | 3 (5.3) | 2 (15.4) |  |
|  | Ghanaian, n (%) | 1 (1.8) | 0 (0.0) |  |
|  | Moroccan, n (5) | 2 (3.5) | 0 (0.0) |  |
| ***Baseline*** | | | | |
| Age, years (mean (SD)) |  | 47.3 (11.0) | 55.2 (10.3) | **0.019**^A^ |
| T2DM, n (%) |  | 11 (19.3) | 3 (23.1) | 0.715^B^ |
| BMI, kg/m^2^ (mean (SD)) |  | 29.2 (6.1) | 28.6 (4.3) | 0.707^A^ |
| Laboratory measurements |  |  |  |  |
|  | Glucose, mmol/L (median (IQR)) | 5.5 (4.9, 6.1) | 5.6 (5.3, 5.9) | 0.821^C^ |
|  | HbA1c, mmol/mol (median (IQR)) | 39 (36, 42) | 42 (35, 43) | 0.667^C^ |
|  | Total cholesterol, mmol/L (mean (SD)) | 5.14 (1.11) | 4.91 (1.38) | 0.518^A^ |
|  | LDL, mmol/L (mean (SD)) | 3.14 (1.12) | 2.92 (0.98) | 0.517^A^ |
|  | HDL, mmol/L (mean (SD)) | 1.30 (0.41) | 1.39 (0.42) | 0.488^A^ |
|  | Triglycerides, mmol/L (median (IQR)) | 1.08 (0.70, 1.82) | 0.77 (0.72, 1.53) | 0.608^C^ |
| ELF-score (mean (SD)) |  | 8.40 (0.58) | 8.98 (0.41) | **0.002**^A^ |
| ***Follow-up*** | | | | |
| Age, years (mean (SD)) |  | 54.2 (11.0) | 62.3 (10.2) | **0.020**^A^ |
| T2DM, n (%) |  | 16 (28.1) | 2 (15.4) | 0.491^B^ |
| BMI, kg/m^2^ (mean (SD)) |  | 29.9 (6.4) | 28.2 (4.8) | 0.361^A^ |
| Waist circumference, cm (mean (SD)) |  | 102.8 (16.7) | 100.0 (13.6) | 0.574^A^ |
| Laboratory measurements |  |  |  |  |
|  | Glucose, mmol/L (median (IQR)) | 6.0 (5.2, 7.1) | 5.6 (5.5, 6.0) | 0.235^C^ |
|  | HbA1c, mmol/mol (median (IQR)) | 39 (35, 48) | 38 (34, 43) | 0.505^C^ |
|  | Total cholesterol, mmol/L (mean (SD)) | 5.07 (1.19) | 5.01 (1.37) | 0.877^A^ |
|  | LDL, mmol/L (mean (SD)) | 3.01 (1.12) | 2.95 (1.05) | 0.851^A^ |
|  | HDL, mmol/L (mean (SD)) | 1.42 (0.39) | 1.49 (0.37) | 0.507^A^ |
|  | Triglycerides, mmol/L (median (IQR)) | 1.17 (0.39, 1.72) | 0.86 (0.79,1.67) | 0.221^C^ |
|  | Platelets, *10^9 (median (IQR)) | 256 (211, 299) | 202 (188, 222) | 0.056^C^ |
| FIB4 (median (IQR)) |  | 1.15 (0.86, 1.51) | 2.04 (1.32, 3.00) | **0.010**^C^ |
| FIB4 categorical | <1.30, n (%) | 30 (52.6) | 3 (23.1) | 0.050^B^ |
|  | 1.30 – 2.67, n (%) | 23 (40.4) | 6 (46.2) |  |
|  | 2.67 – 3.25, n (%) | 2 (3.5) | 2 (15.4) |  |
|  | ≥3.25, n (%) | 1 (1.8) | 2 (15.4) |  |
| ELF (mean (SD)) |  | 8.88 (0.49) | 10.7 (0.92) | **<0.001**^A^ |
| CAP, dB/m (median (IQR)) |  | 299 (245, 345) | 315 (280, 367) | 0.402^C^ |
| CAP categorical | <238 dB/m, n (%) | 12 (21.1) | 1 (7.7) | 0.653^B^ |
|  | 238 – 260 dB/m, n (%) | 6 (10.5) | 1 (7.7) |  |
|  | 260 – 290 dB/m, n (%) | 9 (15.8) | 2 (15.4) |  |
|  | ≥290 dB/m, n (%) | 30 (52.6) | 9 (69.2) |  |
| LSM, kPa (median (IQR)) |  | 6.0 (4.3, 8.6) | 7.3 (5.0, 11.7) | 0.111^C^ |
| LSM categorical | <8.2 kPa, n (%) | 41 (71.9) | 8 (61.5) | **0.028**^B^ |
|  | 8.2 – 9.7 kPa, n (%) | 4 (7.0) | 1 (7.7) |  |
|  | 9.7 – 13.6 kPa, n (%) | 12 (21.1) | 2 (15.4) |  |
|  | ≥13.6 kPa, n (%) | 0 (0.0) | 2 (15.4) |  |

**Supplementary table 2: Participants characteristics at baseline and follow-up of participants stratified for ELF at follow-up.** Participants characteristics at baseline and follow-up visit of participants with ELF <9.8 at baseline, stratified for ELF ≥9.8 at follow-up (n=70 participants). T2DM = type 2 diabetes mellitus; BMI = body mass index; HbA1c = hemoglobulin A1c; IQR = interquartile range; SD = standard deviation; LDL = low-density lipoprotein; HDL = high-density lipoprotein; FIB4= fibrosis-4 score; ELF = enhanced liver fibrosis-score; CAP = controlled attenuation parameter; LSM = liver stiffness measurement. ^A^ = Unpaired t-test; ^B^ = Chi-square test; ^C^ = Mann-Whitney U-test.

| Biomarker at baseline | ELF at follow-up <9.8 (n=57 participants) | ELF at follow-up ≥9.8 (n=13 participants) | p-value |
| --- | --- | --- | --- |
| TNC (median (IQR)) | 12.99 (10.17, 15.72) | 13.77 (12.29, 14.91) | 0.551 ^C^ |
| FBN1 (median (IQR)) | 30.28 (20.84, 38.76) | 20.87 (13.16,58.35) | 0.419 ^C^ |
| Sema4D (median (IQR)) | 0.52 (0.45, 0.63) | 0.58 (0.53, 0.62) | 0.218 ^C^ |
| ADAMTS (median (IQR)) | 25.34 (17.69, 32.94) | 22.70 (12.72, 28,28) | 0.377 ^C^ |
| PAM (median (IQR)) | 64.25 (55.63, 75.33) | 62.59 (54.59,68.43) | 0.511 ^C^ |
| Ssc5D (median (IQR)) | 2.33 (1.41, 3.77) | 3.06 (1.71, 4.26) | 0.154 ^C^ |
| VCAN (median (IQR)) | 103.35 (92.40, 109.65) | 97.65 (93.85, 110.25) | 0.597 ^C^ |
| Urokinase (median (IQR)) | 734.35 (656.05, 854.24) | 757.81 (702.86, 824.62) | 0.910 ^C^ |
| THBS1 (median (IQR)) | 28.60 (20.87, 31.20) | 21.25 (19.93, 25.57) | 0.127 ^C^ |
| IGFBP7 (median (IQR)) | 192.81 (173.34, 207.86) | 219.97 (207.55, 243.63) | 0.001 ^C^ |
| CXCL10 (median (IQR)) | 164.80 (128.10, 182.44) | 201.25 (155.84, 259.64) | 0.071 ^C^ |

**Supplementary table 3: Biomarkers at baseline stratified for ELF at follow-up.** Biomarkers at baseline stratified for ELF-score ≥9.8 at follow-up (n=70 participants). ELF = enhanced liver fibrosis-score; IQR = interquartile range. ^C^ = Mann-Whitney U-test.

| Biomarkers at baseline | AUC (95% CI) |
| --- | --- |
| TNC | 0.55 (0.40, 0.70) |
| FBN1 | 0.72 (0.35, 0.79) |
| Sema4D | 0.61 (0.45, 0.77) |
| ADAMTS | 0.58 (0.38, 0.78) |
| PAM | 0.56 (0.38, 0.74) |
| Ssc5D | 0.63 (0.37, 0.73) |
| VCAN | 0.55 (0.37, 0.73) |
| Urokinase | 0.51 (0.35, 0.67) |
| THBS1 | 0.64 (0.46, 0.81) |
| IGFBP7 | 0.79 (0.64, 0.94) |
| CXCL10 | 0.66 (0.48, 0.84) |

**Supplementary table 4: Diagnostic accuracy of biomarkers.** AUC of biomarkers for the detection of ELF ≥9.8 in participants with ELF <9.8 at baseline (n=70 participants). AUC = area under the curve; CI = confidence interval.

Supplementary figures

**Supplementary figure 1: Validation FFD-diet induced obesity model.** Percentage of hepatic fibrosis based on histological analysis of Picro Sirius Red staining of ob/ob mice fed Normal Control Diet (NCD), Choline-deficient, L-amino acid-defined, high-fat diet (CDAHFD) or Fast Food Diet (FFD) (A). Gene expression of genes after 2 and 4 weeks of FFD feeding in ob/ob mice (B). Significant gene expression is indicated by bold p-values.
